# Experimental Study of Visual Corona under Aeronautic Pressure Conditions Using Low-Cost Imaging Sensors

**DOI:** 10.3390/s20020411

**Published:** 2020-01-11

**Authors:** Jordi-Roger Riba, Álvaro Gómez-Pau, Manuel Moreno-Eguilaz

**Affiliations:** 1 Electrical Engineering Department, Universitat Politècnica de Catalunya, 08222 Terrassa, Spain; 2 Electronics Engineering Department, Universitat Politècnica de Catalunya, 08222 Terrassa, Spain; alvaro.gomez-pau@upc.edu (Á.G.-P.); manuel.moreno.eguilaz@upc.edu (M.M.-E.)

**Keywords:** partial discharge, corona effect, arc tracking, imaging sensor, high voltage, low pressure, more electric aircraft, aeronautics

## Abstract

Visual corona tests have been broadly applied for identifying the critical corona points of diverse high-voltage devices, although other approaches based on partial discharge or radio interference voltage measurements are also widely applied to detect corona activity. Nevertheless, these two techniques must be applied in screened laboratories, which are scarce and expensive, require sophisticated instrumentation, and typically do not allow location of the discharge points. This paper describes the detection of the visual corona and location of the critical corona points of a sphere-plane gap configurations under different pressure conditions ranging from 100 to 20 kPa, covering the pressures typically found in aeronautic environments. The corona detection is made with a low-cost CMOS imaging sensor from both the visible and ultraviolet (UV) spectrum, which allows detection of the discharge points and their locations, thus significantly reducing the complexity and costs of the instrumentation required while preserving the sensitivity and accuracy of the measurements. The approach proposed in this paper can be applied in aerospace applications to prevent the arc tracking phenomenon, which can lead to catastrophic consequences since there is not a clear protection solution, due to the low levels of leakage current involved in the pre-arc phenomenon.

## 1. Introduction

Current aircraft architectures include complex systems and technologies, constituting the core equipment necessary for energizing and flying the aircrafts. In a conventional aircraft design, the engines transform the fuel consumed into power, which is mostly used to propel the aircraft. It is widely recognized that several architecture advances, including a suitable selection of the power offtake location, minimization of engine bleed requirements, elimination of gearboxes, or transition towards electrical powered systems, can significantly optimize the power requirements of the engines, thus improving fuel efficiency and minimizing maintenance requirements. The aviation industry is developing the next generation of more electric aircrafts (MEA) with the final objective of the all-electric aircraft (AEA), which are friendlier from an environmental point of view. The goal is to maximize performance and lower weight [1], reducing O&M costs, increasing overall reliability and power density and reducing greenhouse gas emissions, fuel consumption, and system complexity. This goal can be achieved by means of a gradual introduction of lighter and more compact electrical systems to substitute onboard mechanic, pneumatic, and hydraulic systems [1] used for powering landing gear systems, flight controls, anti-ice systems, brakes, or thrust reversers, as well as to pressurize the cabin or to start the engines. Thus, the next generations of aircrafts will make a more intensive and efficient use of electricity than the predecessors, thus leading to operate at higher voltage levels. Since electrical systems can be switched on only when required, this strategy allows increasing of the efficiency of the whole system. Electrical distribution systems of next generation aircrafts are exposed to greater exigencies due to the increasing complexity and electrical power demanded, jointly with the severe environments at which the components are exposed. The increasing demand for electrical power distribution in aeronautics has led to an increase in the operating voltages in order to maintain the current levels and avoid using heavier wires, thus increasing the electric stress on insulation systems [1,2] in addition to the effects of extreme environmental conditions, i.e., pressure and temperature.

Commercial jet airplanes have typical cruising altitudes around 35,000 feet, i.e., around 10.6 km, although it can vary between 33,000 to 42,000 feet, i.e., 10.1 to 12.8 km, but some private jets can reach altitudes up to 15 km. Therefore, aircraft electrical distribution systems must be highly reliable over a wide pressure range. It is a recognized fact that electrical and electronic assemblies used in aeronautic and aerospace applications are more prone to partial discharge effects due to the low-pressure environments at which they are exposed [3], with corona inception voltages that can be well below those found under normal atmospheric conditions [1]. It is known that at reduced pressure, conductors and electrodes well suited for ground operations can develop corona activity, which is often aggravated by condensation of moisture because of pressure gradients produced during fast airframe ascents and descents. For example, the aircraft industry is developing power systems operating at higher voltage levels, i.e., with bus voltages in the range of 1.5 kV DC up to 4.5 kV DC, thus giving rise to harmful effects, such as corona and arc tracking effects [4]. The increased voltage levels, jointly with the reduced pressure, promote partial discharge inception conditions in insulation systems [2]. Therefore, partial discharge risk must be limited while dealing with systems that are as light and compact as possible [1].

Design engineers must take into account the effect of varying the pressure–distance product on the corona inception and extinction voltages during the lifespan of the electrical and electronic components, including the electrical wires. By means of suitable designs, the reliability of such components can increase notably, reducing the aging, unexpected and unscheduled failures, while easing maintenance plans [3]. Many efforts are being made to design aeronautic power systems for operating at higher voltages. It is imperative to know the conditions at which a corona appears in order to minimize the impact of this harmful effect, since the suitable design of electrical distribution systems requires accurate predictions of the corona inception voltage considering the low-pressure effects. Therefore, experimental work is required to understand and determine the impact of low-pressure environments on corona behavior because it has a notorious effect on the performance of electrical and electronic systems and, if not controlled, can lead to more severe consequences such as arc tracking, which is a type of spark breakdown.

An important part of the radiation is associated with corona falls within the ultraviolet spectrum [5] and the blue visible spectrum, so special care must be taken to detect this effect, particularly at the very early stage. A corona effect is often detected by means of specialized techniques, which often are very expensive, comprising electromagnetic radiation or audible noise [6], such as radio interference voltage (RIV) receivers, PD (partial discharges) detectors, UHF (Ultra High Frequency) sensors [7], optical spectrophotometers [8], or sound level meters [9]. However, by using such sensors, it is not always easy to locate the exact corona points.

Environmental pressure, geometry of the electrodes, supply frequency, and the voltage applied are among the most influential parameters that determine the corona onset conditions. Aeronautic systems must present improved reliability with respect to other applications. Therefore, an exhaustive understanding of the interrelation between high voltage and electrical insulation behavior under low-pressure environments is essential to optimize the behavior and safeguard the integrity of the electrical and electronic components requiring increased reliability. To this end, there is a pressing need to investigate methodically low-pressure effects on the corona inception conditions since there are few research works analyzing corona at low pressures [10], with most of the works in this field studying the disruptive spark breakdown. However, under non-uniform field conditions, a corona may appear at voltage levels well below the disruptive breakdown condition, as shown in this paper.

This work measures the corona extinction voltage and locates the critical corona points of a sphere-plane gap configuration by means of a miniature low-cost sensor, the Sony IMX29 digital camera, which allows detection of the visible as well as part of the UV spectrum. The use of this low-cost sensor, sensitive to both the visible and UV spectra, allows detection of the corona effect in its early stage, while locating the critical points so remedial actions can be taken to prevent a major system failure. This approach allows detection of the discharge points, thus significantly reducing the complexity and costs of the instrumentation required while preserving the sensitivity and accuracy of the measurements. The presented data are based on different pressure conditions ranging from 100 to 20 kPa, thus covering the pressures typically found in aeronautic environments, while different air gap distances are analyzed. Most of the studies refer to power lines operating at higher altitudes [11,12,13], usually below 4000 m, dealing with pressure levels different than those found in aeronautic applications, whereas many other studies are based on spark [14,15,16], glow discharges [17], or discharges in gases other than air [18]. Therefore, there are a lack of experimental studies centered on the inception of corona for aeronautic applications, and this paper makes a contribution to this area. The solution proposed in this work can be applied in aerospace environments to prevent arc tracking activity, where the existing protection systems are unable to detect this phenomenon since, due to the low levels of leakage current involved during the early stage of the phenomenon, they cannot trip conventional thermal-magnetic circuit breakers, thus being undetected, and their effects can be very destructive because of the long-duration overheating of wire insulation.

The rest of the article is organized as follows. Section 2 explains the corona effect and highlights the importance for its premature detection, especially in the case of aeronautics industry. Section 3 describes the effect of pressure in corona inception and breakdown, emphasizing the importance of Paschen’s law. Section 4 presents the experimental setup and the way experimental corona measurements have been carried out under aeronautic pressure conditions. Section 5 presents the obtained experimental results and its discussion. Finally, Section 6 concludes the paper.

## 2. Corona Effect and Arc Tracking

A corona is a type of partial discharge occurring in a gaseous dielectric, i.e., a discharge producing incomplete electrical breakdown of the interelectrode gap, which occurs under non-uniform field conditions whose strength is not sufficient to produce electrical breakdown [19]. Corona discharges take place when the field at the outer surface of a high-voltage electrode is beyond a critical value [20,21,22] since the molecules of the gaseous medium are likely to be ionized [23] because of collisions with electrons, thus initiating a cascade or avalanche of electrons under the influence of an intense electric field which is capable of accelerating the generated free electrons to ionization speeds. It is believed that the initial electrons can be generated by cosmic rays and energetic photoelectrons, which are more abundant and energetic at high altitudes. Solar cosmic rays are rich in neutrons. When these neutrons impact with oxygen and nitrogen molecules, the collision produces a neutron and secondary cosmic rays. Aeronautic and aerospace environments are more affected by cosmic rays that at sea level. For example, at a typical airliner altitude of about 11,000 m, the relative neutron flux is around 390 times higher than at sea level [24]. Therefore, the dielectric strength of air at high altitudes is reduced with respect to that at sea level. However, a detailed description of the whole electrophysicochemical process is a challenging problem which is still not fully described [25].

In air, a corona generates ozone and nitrous acid, carbonizes different types of liquid and solid insulating materials, oxidizes rubber, and corrodes different metal types. Corona activity tends to degrade insulating materials, mostly due to the fast oxidation [26] induced by high-energy species and the ozone generated during the discharge process, which can increase the electrical conductivity of the insulating material and compromise their performance. Electrical discharges also produce electromagnetic radiation, covering visible light, UV, and high-energy ionizing radiation [27]. Partial discharges themselves are among the primary sources of electrical insulation degradation [28] which can lead to early insulation failure in power cables [29]. Thus, coronas can produce fast acceleration of the natural aging process of different electrical and electronic systems, having a negative impact on the reliability of some critical electric and electronic systems. Most of the corona-related problems are produced in cables, wires, and connectors. Coronas produce a gradual erosion of dielectric, leading to eventual micro-arc formation—known as arc tracking—due to the self-sustainment of an arc between two or more wires through a conductive path formed as a result of degradation of the insulation [30].

It is a recognized fact that although the surface field has a key role in corona inception, it does not entirely characterize the corona inception voltage (CIV), since factors such as gas properties or the electric field decay rate away from the electrode surface are also significant factors [31]. Therefore, it is mandatory to develop fast and reliable testing methods to optimize the design of electrical distribution systems, focused to suppress or at least minimize partial discharge occurrence. The design of such systems is more challenging when operating at high altitudes because both corona and flashover inception voltages in air tend to decrease with the operating pressure [13,32,33], thus increasing the risk of insulation failure [26]. The CIV decreases when reducing air pressure or raising temperature because of the expansion of the ionization zone owing to an increase of the effective ionization coefficient [33]. In [19], it is shown that the discharge or ionic current increases as the pressure decreases, which is attributed to a reduced neutral species density (which is proportional to the existing pressure) because of the low-pressure environment, which can be seen as the moving ions offering lower resistance. In addition, the inception voltage decreases with pressure since the primary electrons gain more energy due to the reduced number of collisions with scarcer neutral species and, thus, ion motion depends on the E/N ratio, i.e., the ratio between the electric field strength and the gas density. By means of an ultraviolet camera, [34] concluded that the corona discharge volume around a high-voltage electrode decreases with the atmospheric pressure since corona only can take place in a critical volume in which the ratio E/N is beyond a critical value.

Visual corona tests are often based on the corona extinction and inception voltages. The corona inception voltage, or CIV, is defined as the lowest voltage at which a continuous corona discharge appears when progressively increasing the applied voltage. Conversely, the corona extinction voltage, or CEV, is defined as the highest voltage at which the continuous corona discharge disappears when progressively reducing the voltage applied starting from further than the corona inception voltage [5]. This paper deals with the CEV value, since it is always lower than the CIV, thus representing the worst condition for corona appearance.

Arc tracking is a phenomenon in which due to the degradation of insulation, a micro-arc may be initiated, sustain itself, and propagate along the electrical wires [35], thus generating more damage to the insulation. It appears as a short circuit between adjacent wires and is a critical issue for aeronautics safety [36] since it generates a risk of fire and explosion [37]. It is also known that the current pulses, due to the arc tracking effect, cover a wide band, so it can disturb communication or control systems due to the electromagnetic coupling with other wires [35].

Therefore, this paper deals with the corona effect, since it represents the pre-arc condition, because the chemical reactions favored by corona processes tend to gradually erode the dielectric insulation of the wires, thus leading to eventual arc tracking phenomenon, i.e., a self-sustained micro-arc discharge between adjacent wires through the conductive path generated because of the chemical changes in the insulation [30].

## 3. The Effect of Pressure and the Paschen’s Curve

### 3.1. The Standard Atmosphere

Pressure and, thus, relative air density (RAD), play an important role in the corona onset voltage. Since the atmospheric pressure depends on the altitude above the sea level, it is important to know the relationships altitude–pressure and altitude–RAD, which are given by the standard atmosphere.

**Figure 1:**
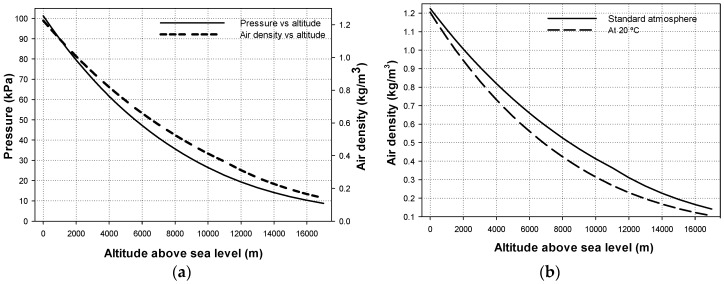
(**a**) Air density and pressure as a function of the altitude above sea level according to the standard atmosphere. (**b**) Air density at standard conditions and extrapolated to 20 °C uniform temperature conditions.

**Figure 2:**
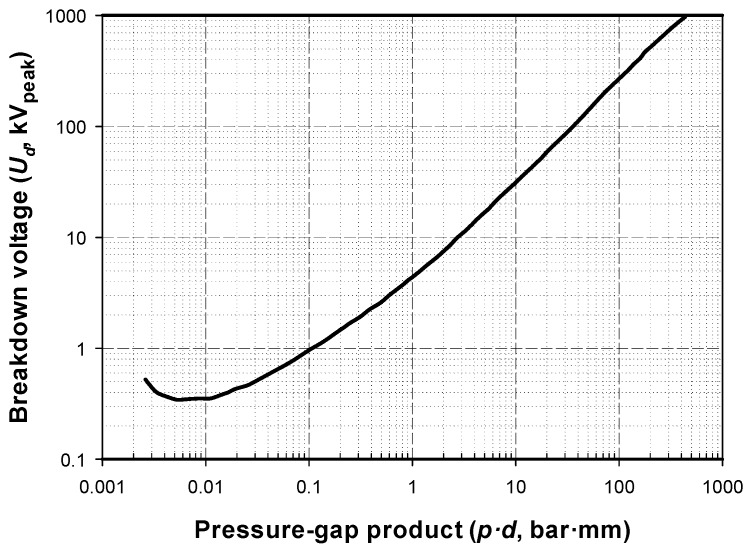
Paschen’s curve for air in uniform field gaps (parallel plates) for air at 20 °C based on experimental data.

Figure 1 and Figure 2 summarize the values of different parameters for air according to the standard atmosphere extracted from the ISO 2533:1975 standard and [38].

### 3.2. Paschen’s Law

Paschen carried out experimental studies of gaseous spark gap breakdown in uniform fields generated between parallel plate electrodes in hydrogen, carbon dioxide, and air at different pressures. These experimental results revealed that the voltage at breakdown conditions depends on the product of the gap length and gas pressure within the gap [39]. However, Paschen’s law only applies for breakdown conditions, neglects space charge effects, and is only applicable to geometries with uniform electric fields. Recently, it has been demonstrated that particle densities (electrons, positive, and negative ions) due to corona-generated space charge increase obviously when decreasing the pressure [40]. Therefore, Paschen curves are not directly applicable to most practical high-voltage situations. For example, most of the practical gaps are non-uniform to some degree, and partial discharges can appear well before a disruptive discharge is formed. However, Paschen’s results are a useful reference since they show a tendency in the behavior of gaseous insulation.

It is known that the breakdown or critical disruptive voltage in gases can be expressed as [41,42]

(1) $$U_{b,peak}=\frac{B\cdot (p\cdot d)}{\ln(A)+\ln(p\cdot d)-\ln[\ln(1+1/\gamma )]}$$

with *A* and *B* being constants whose values depend on the gaseous dielectric, *p* the pressure of gaseous insulation, *d* the gap spacing, and *γ* the secondary ionization or electron emission coefficient.

Figure 2 shows the Paschen’s curve for air at 20 °C based on the work of Dakin et al., which was obtained from experimental data collected from various authors. According to (1) and Figure 2, when dealing with uniform field gaps, the breakdown voltage *U_b_* becomes higher in the low- and high-pressure zones, assuming constant spacing between electrodes [14]. It is noted that for extreme levels of low pressure, i.e., under vacuum conditions, the dielectric strength increases rapidly.

Paschen’s law is of great interest for understanding the constitutive processes in gases and, in particular, for atmospheric air, since it is the insulating medium for most high-voltage applications. This law establishes that the breakdown voltage of a gaseous gap depends on the *p·d* product within the gap (*p* being the pressure and *d* the gap length) instead of *p* and *d* separately [43,44]. When dealing with uniform field gaps, there exists only one curve relating *U_b_* and the *p·d* product. However, other authors have shown that *U_b_* increases with the gap length, even assuming equal values of *p·d*. Therefore, Paschen’s curves for non-uniform gaps with different gap spacing *d* are not superimposed and, instead, they are separately spaced so that the breakdown voltage *U_b_* should be expressed as *U_b_* = *f*(*p,d*) instead of *U_b_* = *f*(*p·d*) [44].

According to the Paschen’s curve for air, the minimum breakdown value lies around 300 V. By supposing *V_min_* ≈ 300 V, that corresponds to the product (*p·d*)_min_ ≈ 6 mbar·mm = 0.61 kPa·mm, and assuming *T* = −56.5 °C and *P* = 22.63 kPa at 11 km altitude above sea level, the gap length at the minimum of the Paschen curve is *d_min_* ≈ 0.027 mm, with this value being about 4.5 times lesser than at sea level. Therefore, the dielectric strength of air at low pressure is much less and, hence, spark discharges are more likely to occur when increasing the applied voltage. This fact dramatically restricts the voltage interval leading to a well-defined unipolar corona discharge [45].

Breakdown voltage values almost do not depend on voltage waveform, i.e., AC, DC, or positive and negative impulse 1/50 µs. However, different factors influence corona onset conditions, including the shape and frequency of the voltage waveform or the type of insulation in the electrodes.

Experiments carried out in this work clearly demonstrate that when reducing the pressure, it becomes more difficult to distinguish between corona and a complete spark discharge or arc. The current of such arcs can be very low. Therefore, when the arcs do not reach the minimum threshold current to trip conventional magnetic/thermal circuit breakers, they go undetected. As a result, the arcs can be very harmful due to long-duration overheating of the polymeric wire insulation, generating a conductive layer of char, a solid residue rich in carbon [46].

It is also stated that frequency changes within the range 60–400 Hz produce no appreciable deviations in the corona onset voltages in capacitors, air gaps, and cables, and they will probably produce insignificant changes in the corona onset voltage for any electrode configuration.

## 4. Experimental Setup

### 4.1. The Low-Pressure Chamber and the Faraday Cage

This work analyzes the CEV pattern of a sphere-plane gap under different pressures, from 100 kPa down to 20 kPa, corresponding to sea level up to 12,000 m altitude, respectively. To this end, a cylindrical low-pressure hypobaric chamber is used jointly with a vacuum pump to reduce the pressure from 100 kPa down to 20 kPa.

The 18/10 stainless steel cylindrical chamber is equipped with a manometer to read the inner pressure. It has a diameter of 260 mm and a height of 375 mm, with these dimensions being enough to conduct the corona tests and to accommodate the required instrumentation.

A one-stage vacuum pump model Bacoeng BA-1, allowing a free air displacement of 3.6 cubic feet per minute and an ultimate vacuum pressure of 0.8 Pa was used to lower the pressure inside the vacuum chamber.

**Figure 3:**
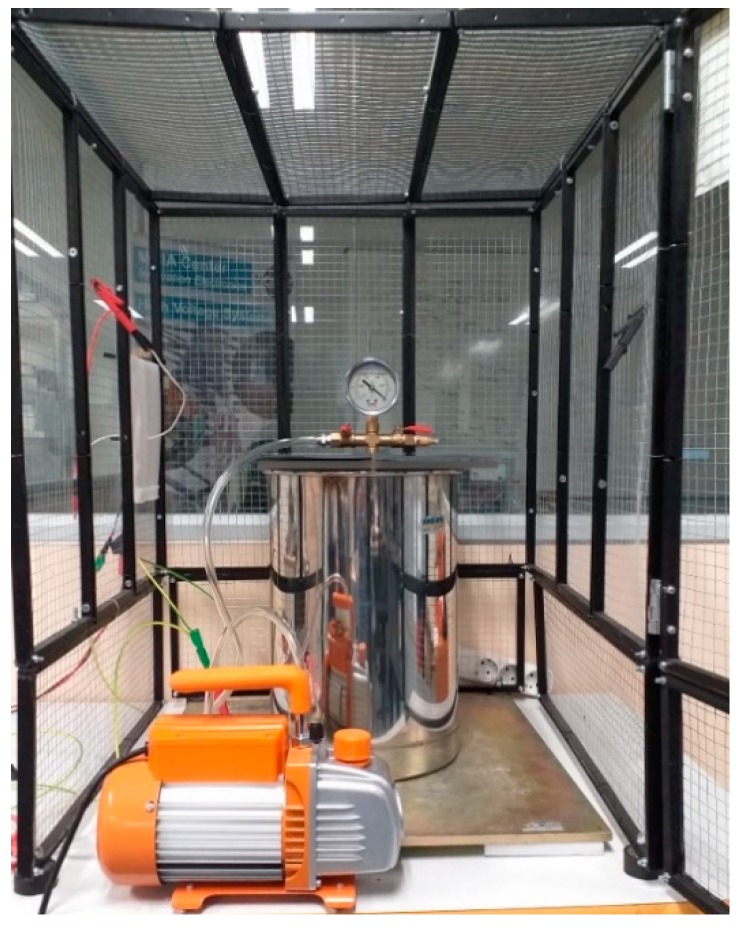
Low-pressure chamber and vacuum pump used for the experiments.

As seen in Figure 3, the cylindrical low-pressure chamber was placed inside a grounded Faraday cage in order to protect both the operators and the equipment from possible electrical shocks.

### 4.2. The Sphere-Plane Electrode and the Supporting Structure

As explained, this paper analyzes the corona behavior of the sphere-plane electrode within the pressure range 100–20 kPa. The sphere has a diameter of 4 mm and the analyzed gap distances (between the bottom part of the sphere and the plane) are 20, 30, and 40 mm, respectively.

**Figure 4:**
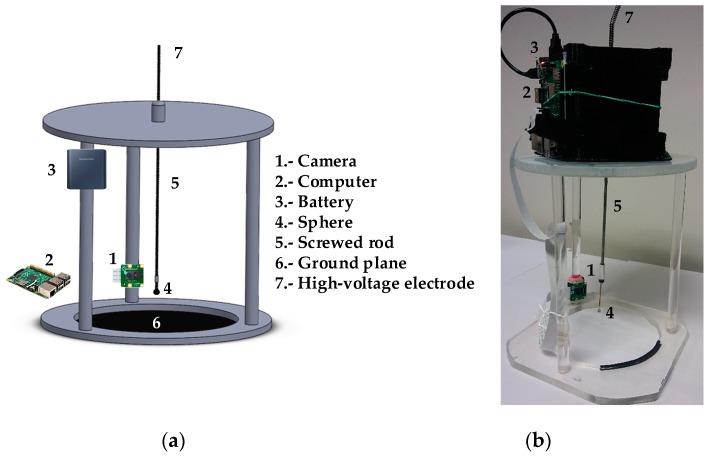
(**a**) Structure to hold the screwed rod supporting the sphere, the imaging sensor, the raspberry pi computer and the battery pack (the wiring between the battery pack, raspberry pi and sensor is not shown). (**b**) The actual constructed structure and the equipment.

To easily change the gap distance, a special plastic structure was manufactured, which is shown in Figure 4. The stainless steel sphere is attached to a 4 mm screwed rod which allows adjusting the gap distance between the bottom of the sphere and the flat ground plane. Both the sphere and the plane are made of stainless steel. The plastic structure also holds the CMOS imaging sensor, the small single-board raspberry pi computer, and the battery pack used to power both the camera and the raspberry pi, which is wirelessly connected to an external computer which receives the data.

### 4.3. The High-Voltage DC Sources

Two high-voltage direct current (DC) power sources were used to supply the sphere-plane gap. The basic data of these sources are found in Table 1.

### 4.4. The Low-Cost 8 MP CMOS Imaging Sensor

Imaging techniques focused on the UV spectrum are being applied in a wide variety of scientific and technical applications, such as remote sensing, fault inspection for industrial applications, or in medical applications, among others. Although UV cameras with high quantum efficiencies are commercially available, they are relatively expensive and can require a relatively high electrical power since some models incorporate a thermoelectric cooling unit. The widespread use of back-illuminated complementary metal oxide semiconductor (CMOS) sensor technology and the important cost decrease of this technology of sensors has made it possible to develop low-cost cameras which are incorporated in consumer electronics, such as smartphones [47]. Back-illuminated CMOS sensors are advantageous over conventional CMOS sensors since they apply a new arrangement of the photodiode to increase the amount of light captured, and thus to improve performance under low light conditions, particularly in the UV spectrum for which these photodetectors are sensitive [48]. This work uses a low-cost Sony IMX29 back-illuminated CMOS camera, specifically designed for the smartphone market, with a cost around 28 euros. The Sony IMX29 camera has a sensor resolution of 3280 × 2464 pixels (8 MP), a sensor image area of 3.69 mm × 2.81 mm, a pixel size of 1.12 µm × 1.12 µm and an optical size of 1/4, and allows video modes of 1920 × 1080 pixels (up to 30 fps), 1280 × 720 pixels (up to 90 fps), and 640 × 480 pixels (up to 90 fps). To improve sensitivity and color reproducibility, it is required that sensor data is retrieved in RAW format [48].

It is widely recognized that backside-illuminated CMOS sensors are also sensitive to UV, so that the incident irradiation on the photodiodes is converted into an electric current, its magnitude depending on the spectral sensitivity response of the photodiode and the incident irradiation intensity [49].

**Figure 5:**
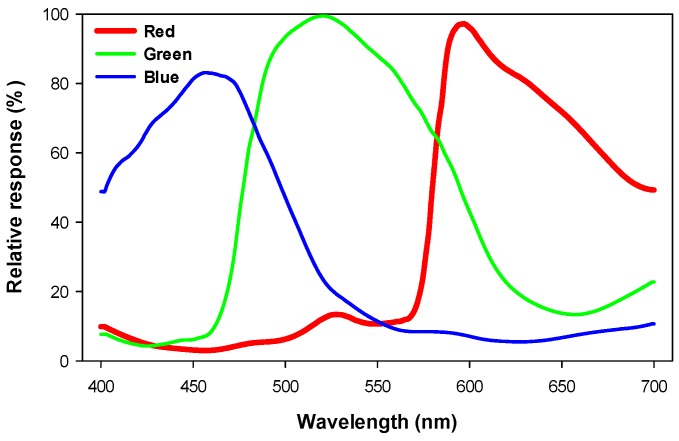
Spectral response curves of the Sony IMX29 camera [50].

Figure 5 shows the spectral response curves of the Sony IMX29 camera.

### 4.5. The Wireless Communication through the Raspberry Pi Computer

The image data acquired by the 8 MP CMOS imaging sensor commanded by the raspberry pi computer must be wirelessly sent to an external computer for further processing and analysis. This procedure allows the precise determination of the exact CEV point using the algorithm described and listed in Section 5. The raspberry pi computer runs a Raspbian Linux distribution in which two Python 3 scripts have been developed in order to manage the captures of the low-cost CMOS imaging sensor. The former is in charge of showing a real time preview of the sphere within the vacuum chamber and, therefore, a real time visual corona determination can be achieved while the latter one adequately configures the capture options to take a long exposure photograph which will be further processed as explained in the next section.

### 4.6. The Image Processing Step

**Figure 6:**
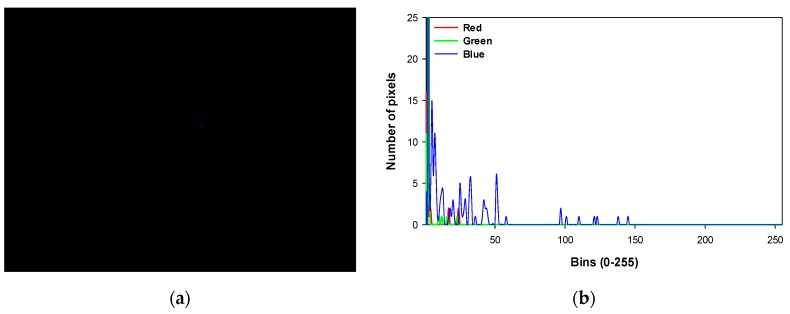
(**a**) Capture of the corona effect close to its extinction voltage. Note the almost indistinguishable blue pixels at the center of the image. (**b**) The color histogram of the previous capture, identifying the presence of very few pixels with high blue intensity (0 to 255 range).

Each of the long exposure photographs transferred to the computer must be processed in order to determine if the corona effect has arrived at its extinction point or, on the contrary, is still present. In order to achieve such a goal, a histogram of the captured image is performed for each of the three color channels (red, green, and blue). With the aid of the color histogram, the determination of the existence of corona becomes a systematic task. See, for instance, Figure 6, which shows an image with very little corona, which may be difficult to assess with a naked eye, although the color histogram is capable of identifying a considerable number of high intensity blue pixels.

**Figure 7:**
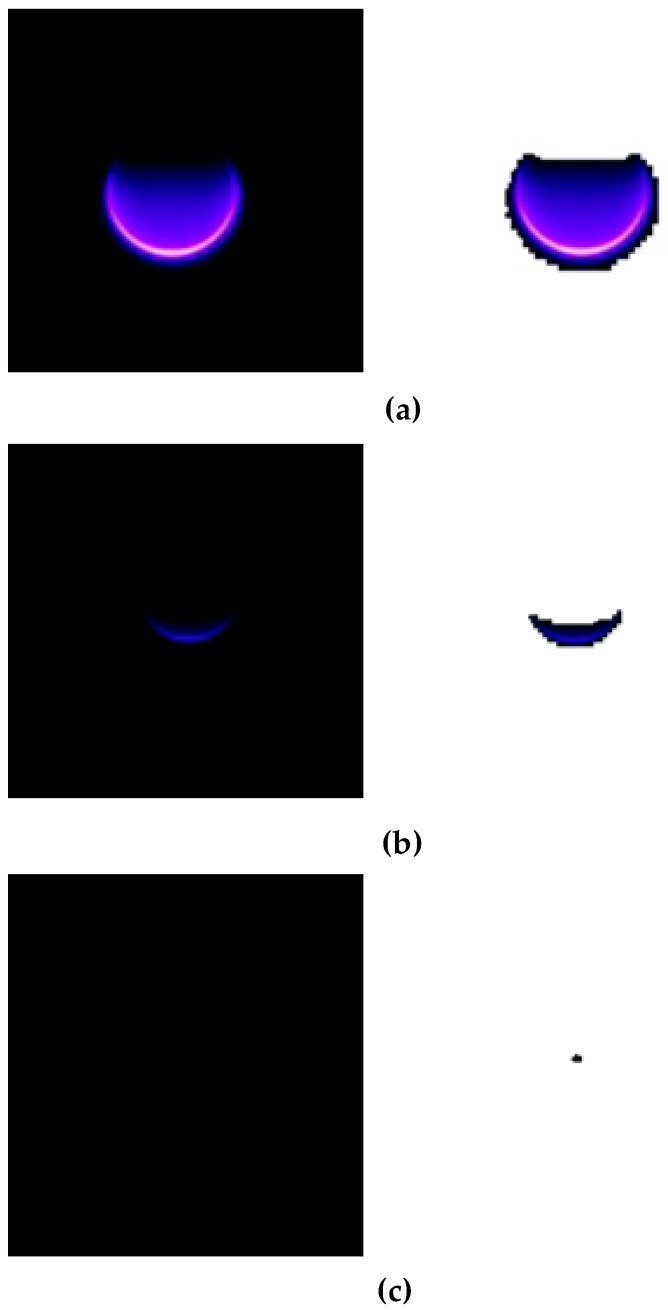
Corona photographs under positive DC supply, air gap = 30 mm, 50 kPa. (**a**) 14.2 kV; (**b**) 8.6 kV; (**c**) Corona extinction voltage, 8.4 kV.

**Figure 8:**
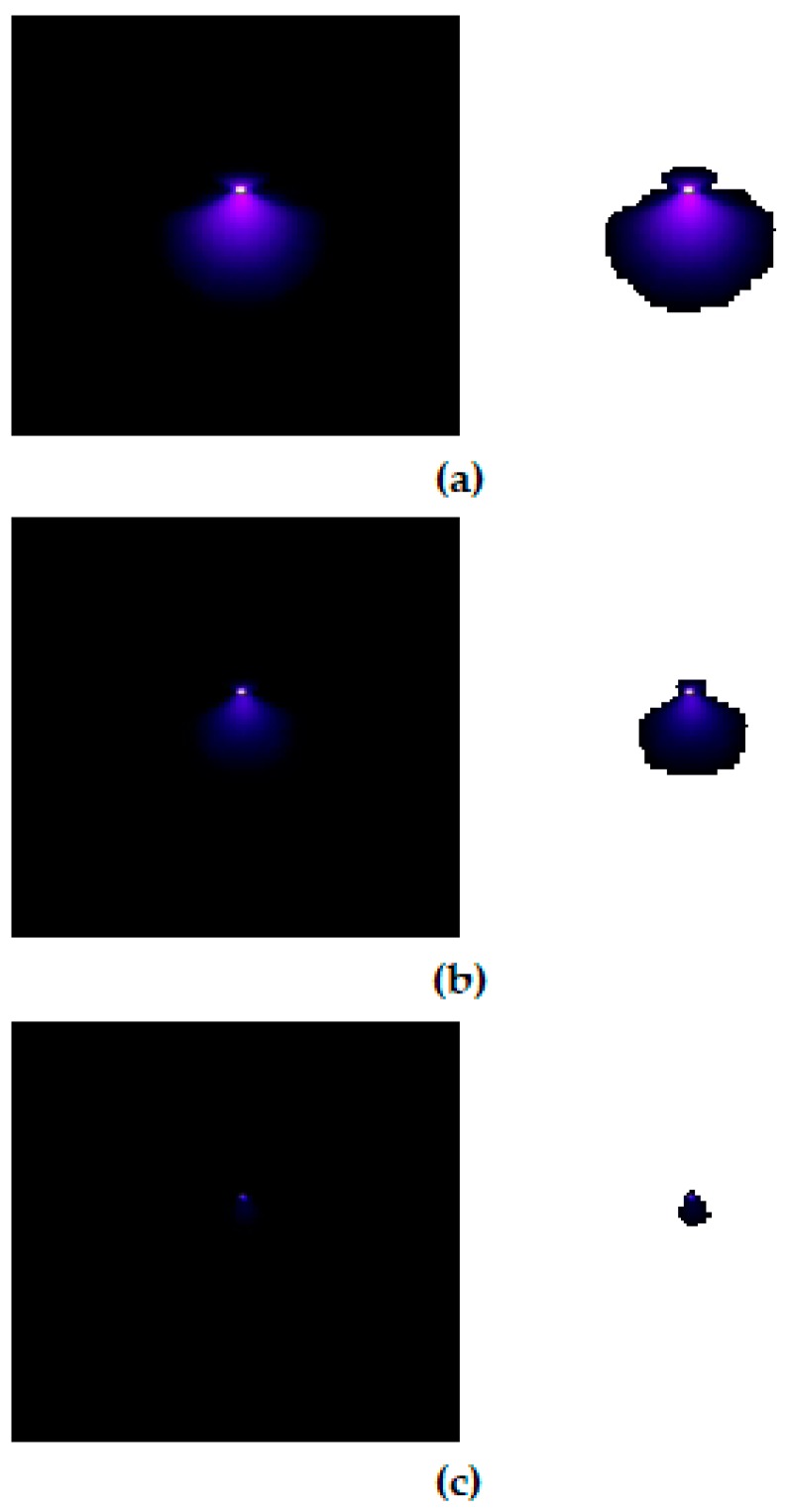
Corona photographs under negative DC supply, air gap = 40 mm, 40 kPa. (**a**) 8.5 kV; (**b**) 7.9 kV; (**c**) Corona extinction voltage, 7.6 kV.

In order to assess the presence of corona with even more confidence, an extra image processing step is carried out. It consists of the transformation of all black color pixels to pure white color in order to endow more contrast to the corona capture and facilitate the detection of corona evidence. The transformation is a fuzzy transform within a 2% tolerance for blacks. Examples of such contrasting procedure are seen in Figure 7 and Figure 8.

## 5. Results and Discussion

This section summarizes the experimental results attained when supplying the 4 mm diameter stainless steel sphere with stabilized positive DC and negative DC voltages. All tests were carried out inside the low-pressure chamber. The procedure of the tests was as follows:

1. Increase the voltage from 0 V to some kV at a rate of 1 kV/s in order ensure stabilized corona conditions. Next, decrease the voltage in some extent while ensuring the corona is still present and take a long exposure photograph (exposure time = 10 s, ISO = 800). It is possible to check the corona effect by previsualizing the video image provided by the Sony IMX29 camera.
2. Decrease the voltage once again to some extent, and take a long exposure photo to ensure there is corona.
3. If there is still corona in Step 2, decrease the voltage again and take another long exposure photograph, otherwise, the CEV is the average value of the voltages recorded in Steps 1 and 2.

The abovementioned process is repeated until the corona disappears. Figure 7 and Figure 8 show the sequence of the long exposure corona photographs taken by the Sony IMX29 back-illuminated CMOS camera according to the procedure detailed above, under positive and negative DC supply, respectively.

**Figure 9:**
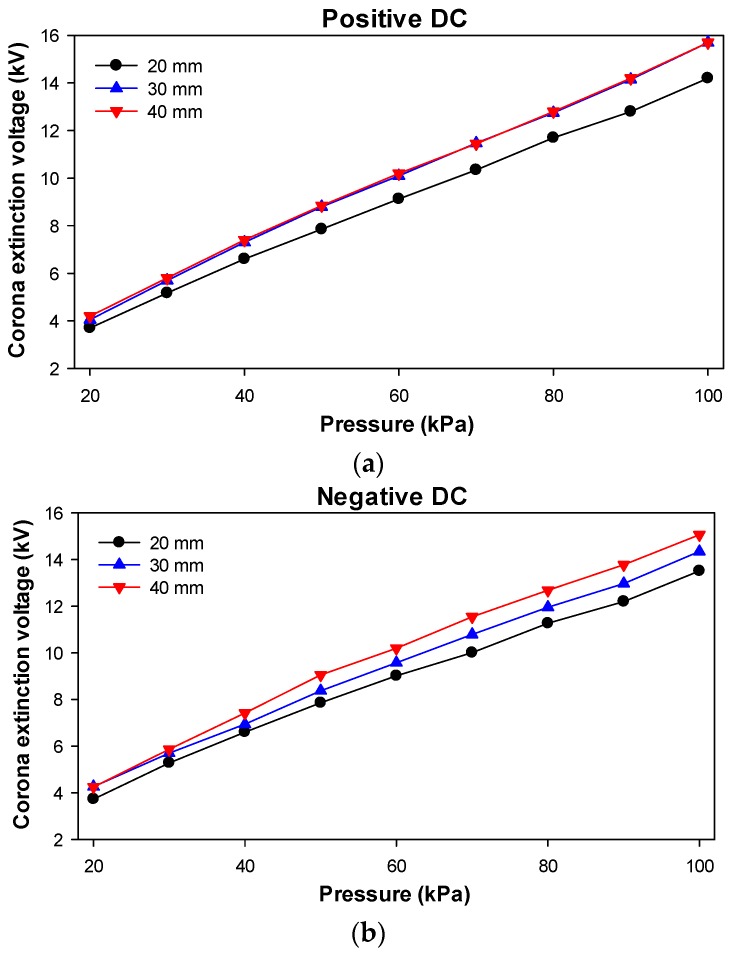
Corona extinction voltage (CEV) values versus air pressure. (**a**) Positive DC supply; (**b**) Negative DC supply.

Figure 9 shows the CEV values recorded at the different heights of the sphere above the ground plane (20, 30, and 40 mm) versus the pressure of air.

The results provided in Figure 9 show an almost linear behavior of the CEV values versus the pressure, for both positive and negative DC polarities of the applied voltage.

Table 2 summarizes the linear dependencies of the CEV versus the pressure of air, showing a very linear and thus predictable behavior. These results show a slightly higher slope of the CEV versus the pressure under positive DC supply, although at 100 kPa, the CEV values are lower under negative DC supply, as stated in the technical literature [22].

**Figure 10:**
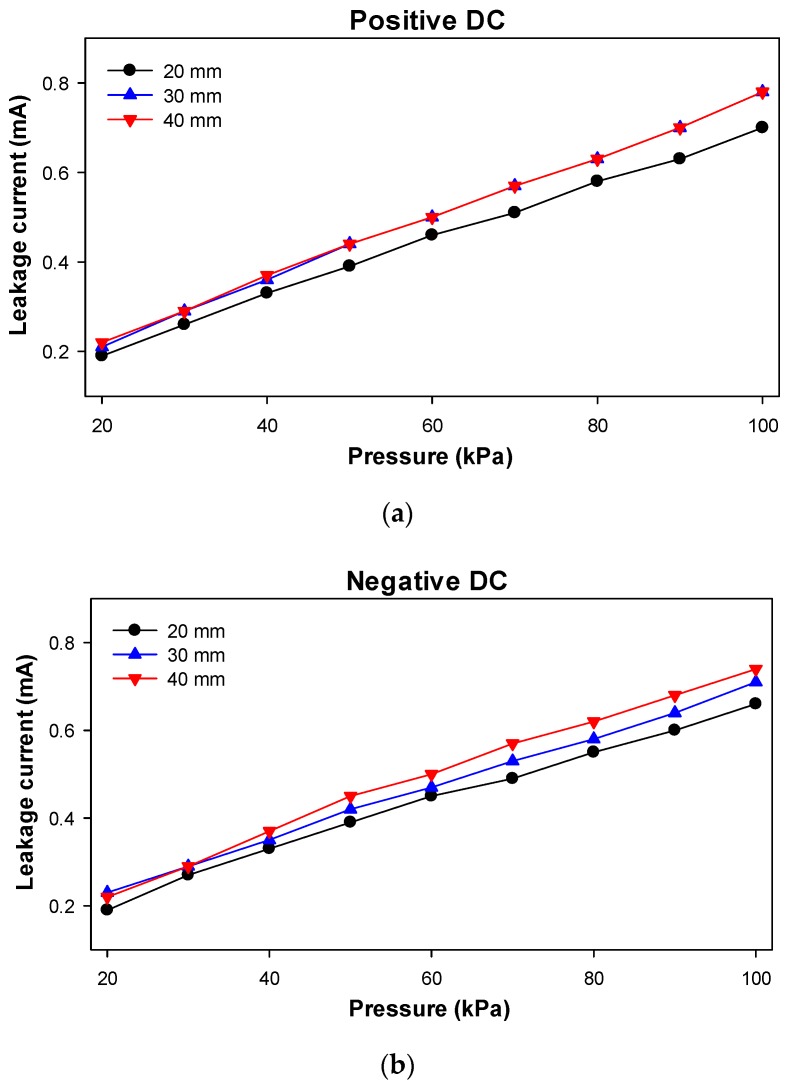
Leakage current versus air pressure. (**a**) Positive DC supply; (**b**) Negative DC supply.

The leakage currents were also measured during the experiments, since they provide information about the ionization process. These results are summarized in Figure 10 and Table 3.

The results presented in Figure 10 and Table 3, related to the leakage current versus the operating temperature, show almost the same tendencies as compared to CEV versus pressure results presented in Figure 10 and Table 2. Therefore, it can be concluded that CEV and leakage current are directly related.

**Figure 11:**
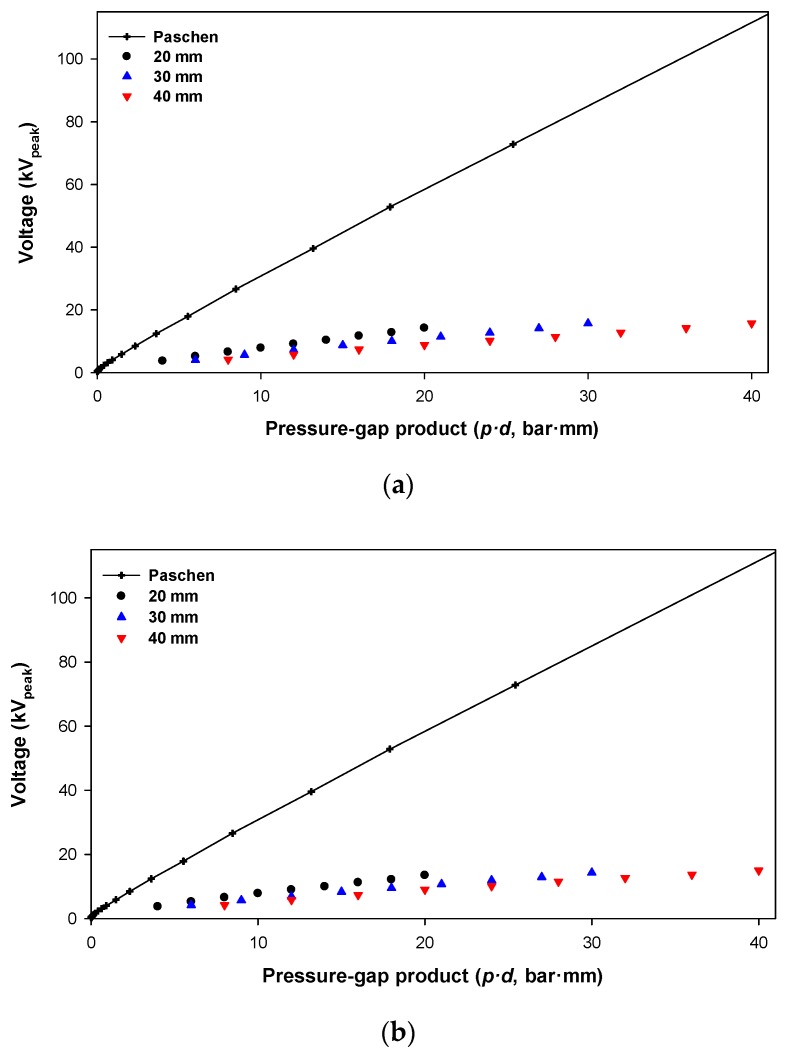
(**a**) Positive DC supply; (**b**) Negative DC supply.

Finally, it is important to determine the relationship between Paschen’s curve for air and the results obtained in this work. This comparison is summarized in Figure 11.

Results from Figure 11 clearly show that the breakdown voltage according to the Paschen’s curve for air is much beyond the CEV, with this difference depending on the configuration of the experimental setup (20, 30, and 40 mm gap). Therefore, the CEV value is much more limiting than the values of the Paschen’s curves, so full consideration must be given to the CEV value when designing high-voltage systems for aerospace applications, although the difference between both values tends to vanish at very low *p·d* values.

## 6. Conclusions

Due to the increment of electrical power installed in more electric aircrafts, the tendency is to increase the bus voltage at levels much higher than the current ones. These higher voltage levels and the environmental conditions typical of aeronautic applications, in particular the reduced pressure, promote partial discharge and corona occurrence in insulation systems. Therefore, there is a pressing need to detect such harmful effects in the early stage by using suitable sensing systems. Among the standard systems to detect corona activity, the partial discharge and radio interference voltage measurements are highlighted, although such methods require very low levels of electromagnetic noise, thus requiring screened facilities, sophisticated, and very expensive and bulky instrumentation, whereas these measurements typically do not allow for location of the exact discharge points.

This paper has applied a low-cost Sony IMX29 back-illuminated CMOS camera to detect the corona effect under aeronautic pressure conditions, specifically in the range 100 kPa down to 20 kPa. It is noted that this very small sensor is sensitive to both the visible and UV spectrum, so that it takes advantage of the light emitted by the corona effect, and it can be placed in many locations because of its small size, while allowing identification of the exact location of the discharge points and the extent of the corona fault condition.

To this end, a sphere-plane gap has been analyzed, where three gap distances have been studied under both positive and negative DC supply. Experimental results presented in this paper show both the corona extinction voltage and the leakage current as a function of the working air pressure. Such results have determined that the CEV–pressure and leakage current–pressure relationships are very linear for both positive and negative DC supply, so the leakage current can be regarded as a good indicator of the corona activity. It has also been demonstrated that when designing high-voltage systems for reduced pressure applications, the CEV value must be considered as a reliable limit instead of the breakdown voltage in the Paschen’s curves, although the difference between both values reduces when decreasing the *p·d* value.

It has been demonstrated that the use of this low-cost sensor is appealing since it allows detection of corona activity in the early stage and identification of the critical corona points, without the need to perform prior calibration. This sensor is also easy to install in different locations due to its reduced size and it can be placed very close to the parts or devices susceptible to generating this harmful effect. This sensing method also allows a drastic reduction in the complexity and cost of the instrumentation required for corona detection while simplifying the requirements of the measurement process.

Finally, the method suggested in this work can be applied in the aerospace sector to prevent the arc tracking phenomenon in its early stage, which is regarded as a destructive phenomenon which ultimately can lead to catastrophic consequences.

## Figures and Tables

**Table 1:** Characteristics of the high-voltage DC sources used in this work.

Characteristics	Source 1 (Positive DC)	Source 2 (Negative DC)
Manufacturer	Phenix Technologies	Phenix Technologies
Model number	4120-10	4120-10
Output voltage	0–120 kV	0 to −120 kV
Output current	0–10 mA	0–10 mA
Voltage ripple	<2% RMS (40 MΩ resistive)	<2% RMS (40 MΩ resistive)
Leakage current measurement	0.02 μA–10 mA	0.02 μA–10 mA

**Table 2:** Main parameters of the CEV versus air pressure linear regression.

	Characteristics	20 mm	30 mm	40 mm
Positive DC	Slope (kV/kPa)	0.1293	0.1425	0.1410
	Intercept (kV)	1.2966	1.4502	1.6067
	*R* ^2^	0.9991	0.9986	0.9988
Negative DC	Slope (kV/kPa)	0.1190	0.1244	0.1334
	Intercept (kV)	1.6941	1.9747	1.9813
	*R* ^2^	0.9968	0.9984	0.9948

**Table 3:** Main parameters of the leakage current versus air pressure linear regression.

	Characteristics	20 mm	30 mm	40 mm
Positive DC	Slope (mA/kPa)	0.0063	0.0070	0.0069
	Intercept (mA)	0.0730	0.0798	0.0880
	*R* ^2^	0.9987	0.9988	0.9990
Negative DC	Slope (mA/kPa)	0.0057	0.0059	0.0064
	Intercept (mA)	0.0957	0.1149	0.1063
	*R* ^2^	0.9959	0.9989	0.9953
